# Characterizing metabolic interactions in a clostridial co-culture for consolidated bioprocessing

**DOI:** 10.1186/1472-6750-13-95

**Published:** 2013-11-04

**Authors:** Fahimeh Salimi, Radhakrishnan Mahadevan

**Affiliations:** 1 Department of Chemical Engineering and Applied Chemistry, University of Toronto, 200 College Street, Toronto, ON M5S 2E6, Canada

**Keywords:** Consolidated bioprocessing, Clostridial co-culture, qPCR analysis, *Clostridium acetobutylicum*, *Clostridium cellulolyticum*

## Abstract

**Background:**

Clostridial co-culture containing cellulolytic and solventogenic species is a potential consolidated bioprocessing (CBP) approach for producing biochemicals and biofuels from cellulosic biomass. It has been demonstrated that the rate of cellulose utilization in the co-culture of *Clostridium acetobutylicum* and *Clostridium cellulolyticum* is improved compared to the mono-culture of *C. cellulolyticum* (BL 5:119-124, 1983). However, the metabolic interactions in this co-culture are not well understood. To investigate the metabolic interactions in this co-culture we dynamically characterized the physiology and microbial composition using qPCR.

**Results:**

The qPCR data suggested a higher growth rate of *C. cellulolyticum* in the co-culture compared to its mono-culture. Our results also showed that in contrast to the mono-culture of *C. cellulolyticum*, which did not show any cellulolytic activity under conditions similar to those of co-culture, the co-culture did show cellulolytic activity even superior to the *C. cellulolyticum* mono-culture at its optimal pH of 7.2. Moreover, experiments indicated that the co-culture cellulolytic activity depends on the concentration of *C. acetobutylicum* in the co-culture, as no cellulolytic activity was observed at low concentration of *C. acetobutylicum*, and thus confirming the essential role of *C. acetobutylicum* in improving *C. cellulolyticum* growth in the co-culture. Furthermore, butanol concentration of 350 mg/L was detected in the co-culture batch experiments.

**Conclusion:**

These results suggest the presence of synergism between these two species, while *C. acetobutylicum* metabolic activity significantly improves the cellulolytic activity in the co-culture, and allows *C. cellulolyticum* to survive under harsh co-culture conditions, which do not allow *C. cellulolyticum* to grow and metabolize cellulose independently. It is likely that *C. acetobutylicum* improves the cellulolytic activity of *C. cellulolyticum* in the co-culture through exchange of metabolites such as pyruvate, enabling it to grow and metabolize cellulose under harsh co-culture conditions.

## Background

In consolidated bioprocessing (CBP) all four biological steps involved in the conversion of cellulosic biomass, which includes production of saccharolytic enzymes, hydrolysis of the polysaccharides present in pre-treated biomass, and fermentation of hexose and pentose sugars present in the hydrolyzate to desired products, takes place in one bioreactor using a single microorganism or a microbial consortium without the external addition of saccharolytic enzymes [1]. Consolidated bioprocessing has been proposed to decrease the production cost by eliminating the costs associated with the cellulase production stage. CBP requires microorganisms with both rapid conversion of cellulose and high product yield, productivities and titres, while such microbes have not been identified in nature yet and need to be developed [2-5]. To realize this aim, two strategies have be applied: the native strategy which improves the product formation capabilities, such as yield and titre in natural cellulolytic microorganisms [6-11], and the recombinant strategy that involves engineering non-cellulolytic organisms with high product yields so that they will express heterologous cellulase and be able to utilize cellulose [1,5,12].

**Figure 1:**
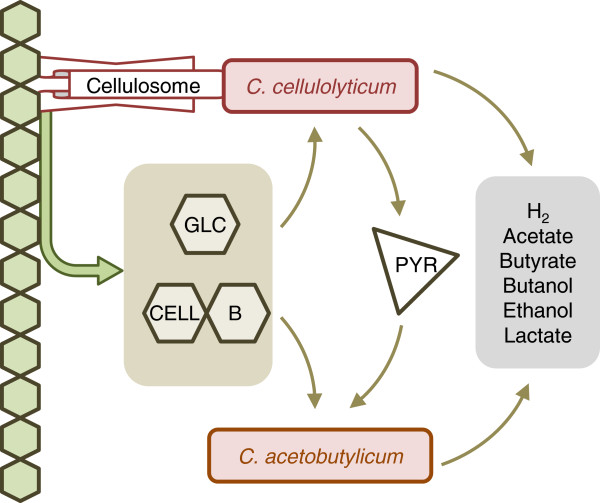
**The scheme of the clostridial co-culture fermentation on cellulose.***C. cellulolyticum* adheres to the cellulose fibers using cellulosome and hydrolyzes cellulose to cellobiose (cellb) and glucose (glc), which can be metabolized by *C. cellulolyticum* and *C. acetobutylicum* in the coculture. The produced pyruvate (pyr) can also be fermented as a carbon source by *C. acetobutylicum.*

An alternative method for the production of biobutanol and biochemicals from cellulosic biomass in a consolidated bioprocessing approach is the use of mesophilic clostridial co-culture. *Clostridium acetobutylicum* shows an effective capability to ferment cellulose derived sugars as well as hemicellulose derived sugars, such as cellobiose, mannose, arabinose, xylose, glucose, and galactose to acetone, butanol, and ethanol [13-16]. Thus, the co-culture of this bacterial species with a mesophilic cellulose degrading bacterium can be an efficient approach. *Clostridium cellulolyticum* is a cellulolytic, mesophilic bacterium which is able to solublize crystalline cellulose in pretreated hardwood [17]. A simplified scheme of this clostridial co-culture fermentation on cellulose is presented in Figure 1. *C. cellulolyticum* synthesizes cellulosome, which is an extracellular multi-enzymatic complex, and degrades cellulose, with the use of this cellulosome, to glucose and soluble cellodextrins (mainly cellobiose), which can be fermented by both species in the co-culture.

The co-culture of *C. cellulolyticum* with *C. acetobutylicum* has been studied previously, and it has been shown that cellulolytic activity is the limiting factor in the co-culture fermentation since most of the cellulase activity products are consumed by *C. acetobutylicum*. The fermentation products have been mainly butyric acid along with butanol, acetic acid and ethanol, and the lack of glucose, which is required for solvent production due to low cellulolytic activity, was hypothesized to be the reason for acid accumulation [18,19]. Furthermore, three times more cellulosic material was degraded in the co-culture compared to the mono-culture of *C. cellulolyticum* due to the utilization of cellulase activity products and the elimination of their repressive effects [20], suggesting the presence of synergism between these two species. The analysis of this effect can be valuable for optimizing the rate of cellulosic material degradation.

Therefore, in this study, we investigated metabolic interactions in this co-culture by developing a comparative qPCR analysis of the co-culture and mono-cultures of *C. cellulolyticum* and *C. acetobutylicum*. Investigation of the metabolism in this clostridial co-culture along with the mono-cultures revealed that significant increase in the rate of cellulose hydrolysis can be achieved using the co-culture and making use of the synergism existing between the two clostridial species. It is likely that *C. acetobutylicum* improves the cellulolytic activity of *C. cellulolyticum* in the co-culture through exchange of metabolites such as pyruvate, enabling it to grow and metabolize cellulose under harsh co-culture conditions. This clostridial co-culture can offer a considerable potential CBP approach for producing commodity chemicals from cellulosic biomass, taking advantage of *C. acetobutylicum* metabolic potential in converting sugars to variety of chemicals.

## Results and discussion

### Experimental characterization of *C. cellulolyticum* cellulolytic activity at high cellulose concentration, addressing the pH effect

**Figure 2:**
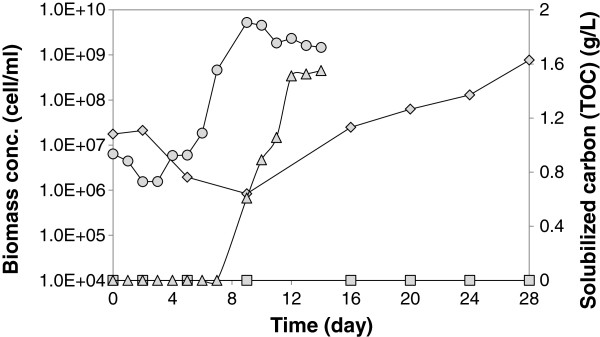
**The total solubilized carbon and biomass profiles in the*C. cellulolyticum*mono-culture experiments.** The biomass profile in the mono-culture at pH of 7.2 (circle), TOC profile in the mono-culture at pH of 7.2 (triangle), biomass profile in the mono-culture under pH profile (rhombus), and TOC profile in the mono-culture under pH profile (square).

In the previous co-culture study [19], the pH effect on *C. cellulolyticum* growth and cellulolytic activity has not been addressed; therefore, to investigate the pH effect on *C. cellulolyticum* growth and cellulolytic activity in a mono-culture at high cellulose concentration of 20 g/L, two batch cultures of *C. cellulolyticum* were conducted: one batch at pH of 7.2, which is an optimal pH for *C. cellulolyticum* growth and cellulolytic activity [21], and another batch with the same pH profile as the co-culture run, i.e. initially at pH of 7.0 for 2 days followed by a pH switch to 6.0. The profiles of cellulose solubilization and biomass concentration are shown in Figure 2. In the mono-culture at pH of 7.2, TOC data showed that 1.5 g/L of carbon was solubilized after 14 days, which is equivalent to 4.9 g/L of cellulose degraded, assuming 24% of carbon flow goes towards CO_2_ formation; this amount of degraded cellulose was comparable to 5.1 g/L reported for the *C. cellulolyticum* mono-culture at the same initial cellulose concentration [19,22]. Also, qPCR data showed that cells reached a stationary growth phase after 9 days with a maximum cell density of 5× 10^9^ cell/mL and a specific growth rate of 1.23 day^-1^.

In contrast, the mono-culture run under co-culture pH profile did not show any cellulose solubilization during the batch (Figure 2), and cells had a very low growth rate of 0.16 day^-1^ (87% decrease) compared to the mono-culture batch at pH of 7.2. The effect of pH on *C. cellulolyticum* growth and cellulolytic activity at low cellulose concentration of 3.7 g/L has been addressed previously [21], and it has been shown that *C. cellulolyticum* is significantly affected by acidic pH, where a pH drop from 7.0 to 6.4 leads to fourfold lower biomass concentration. It has been suggested that acidic pH hampers biomass formation, likely through direct effect of pH on a cellular constituent such as an enzyme or a transport protein, rather than cellulose degradation capability in *C. cellulolyticum*. This argument is supported by the observation that the flux through cellulose degradation reaction remains almost unvarying, in the range of 1.69 to 1.84 mmol (g cell. h)^-1^, while pH varies between 6.4 to 7.0 [21]. However, regardless of the mechanism of inhibition, this pH effect must be considered when comparing cellulolytic activity and *C. cellulolyticum* growth and metabolism in the mono-culture and co-culture.

### Experimental characterization of the co-culture metabolism

**Figure 3:**
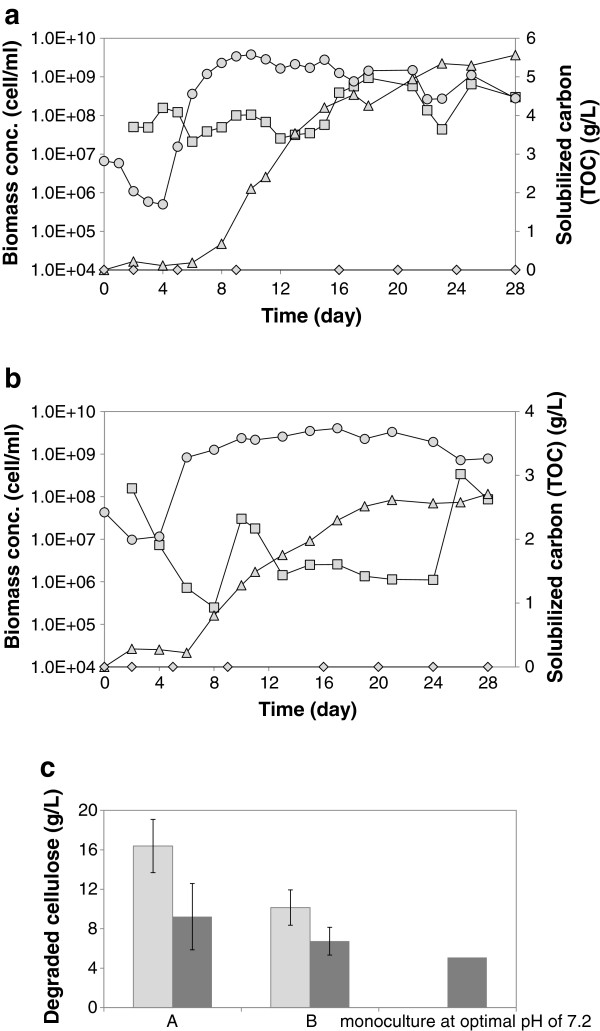
**The cellulose solubilization in the co-culture and mono-culture experiments. (a)** The profiles of cellulose degradation and biomass formations in the co-culture experiment A (with autoclaved iron solution), where figure **(a)** shows one data set representative of two independent experiments, with both showing comparable results; **(b)** The profiles of cellulose degradation and biomass formations in the co-culture experiments B (with filter sterilized iron solution), where figure **(b)** shows one data set representative of two independent experiments, with both showing comparable results. The time profiles of *C. cellulolyticum* (circle), *C. acetobutylicum* (square) and cellulose solubilization in terms of total organic carbon in each co-culture (triangle) at initial cellulose concentration of 20 g/L and under pH profile, along with cellulose solubilization in terms of total organic carbon in *C. cellulolyticum* mono-culture under the same co-culture conditions (rhombus). *C. acetobutylicum* was inoculated after 2 days; **(c)** Degradation of cellulose in the co-culture experiments A and B and the mono-culture experiment at optimal pH of 7.2, 5 days after reaching the maximum *C. cellulolyticum* concentration in the culture (dark grey) and after 28 days (light grey). Error bars are based on two duplicate experiments.

To prepare the co-culture medium for the first co-culture experiment A, all media components including the iron solution were autoclaved. The profile of cellulose degradation in this co-culture is shown in Figure 3a. At initial cellulose concentration of 20 g/L, which is equivalent to 6.08 g/L of final TOC concentration, assuming full degradation of cellulose and considering 24% of the total carbon would be used toward CO_2_ formation, about 82% of cellulose was degraded after 28 days in the co-culture experiment A. Compared to the mono-culture at optimal pH of 7.2, cellulose degradation showed about 82% improvement as shown in Figure 3c, whereas no cellulose degradation was observed in the mono-culture run under co-culture pH profile. These results confirm that *C. acetobutylicum* metabolic activity significantly improves the cellulolytic activity in the co-culture, and in fact makes it possible for *C. cellulolyticum* to survive under harsh co-culture conditions, which do not allow it to grow and metabolize cellulose independently.

Furthermore, to check and ensure iron sufficiency in the co-culture medium, the co-culture medium was prepared by filter sterilizing the ferrous sulphate solution (experiment B), rather than autoclaving as for co-culture experiment A, to avoid potential iron oxidation during medium preparation. The results of this experiment are shown in Figure 3b; while the cellulolytic activity was high in the co-culture experiment B, and *C. cellulolyticum* growth rate was still as high as experiment A, but the cellulose degradation was declined by 38% compared to experiment A, as it is presented in Figure 3c. Cellulose degradation in each batch was estimated from carbon balance and TOC measurements, taking into account that about 24% of the total carbon has been utilized for CO_2_ formation, as described in analysis section. These results confirmed that the co-culture was not under iron limiting condition, and the presence of more ferrous ions had an adverse effect on the co-culture cellulolytic activity.

The mechanism underlying such a synergy between the two clostridial species is not clearly understood. Hence, in order to understand the nature of interactions between *C. acetobutylicum* and *C. cellulolyticum*, that improve cellulose solubilization, we used qPCR to track the population of each species in the batch cultures. Figure 3a and b show the dynamic profiles of each species population in the co-culture batches. *C. cellulolyticum* biomass concentration reached to 3×10^9^ cell/mL in the co-cultures, which was the same value for maximum biomass concentration in the mono-culture runs; however the growth dynamics was significantly faster in the co-cultures compared to the mono-culture run under the same pH profile. *C. cellulolyticum* growth rate in the co-culture was comparable to its growth rate in mono-culture under optimal pH condition of 7.2. Also, it could be observed from all co-culture TOC profiles that cellulose solubilization started after *C. cellulolyticum* had reached its late exponential growth phase.

*C. acetobutylicum* biomass profiles are also shown in Figure 3, where the initial decrease in biomass concentration in the co-culture could be attributed to the lack of available sugars for *C. acetobutylicum* to grow while *C. cellulolyticum* had been in the lag phase. Furthermore, a considerable growth could be observed for *C. acetobutylicum*, when *C. cellulolyticum* had reached its maximum concentration in the co-culture, where possibly more sugars became available in the co-culture to support *C. acetobutylicum* growth. Although *C. acetobutylicum* biomass concentration showed significant increases at some points over the course of co-culture, it was fluctuating and did not remain constant. It has been suggested that in the microbial communities growing on cellulosic material, where there is a competition between cellulose-adherent cellulolytic microorganisms and non-adhered microbes for cellulose hydrolysis products, cellulose-adherent cellulolytic microorganisms are possibly successful winners [2], and this phenomenon could explain the limited growth of *C. acetobutylicum* in the co-culture.

In addition, *C. acetobutylicum* has cellobiase and endoglucanase activities, but is not able to hydrolyze crystalline cellulose due to lack of the required enzymatic activities [23], although it produces cellulosome [24]; therefore, the improved cellulolytic activity in the co-culture cannot be attributed to *C. acetobutylicum* cellulolytic activity. However, *C. acetobutylicum* is able to ferment cellobiose and cellulose-derived sugars, and the improved cellulolytic activity in co-culture can be attributed to the role of *C. acetobutylicum* in consuming sugars and preventing the carbon overflow toward *C. cellulolyticum*, as *C. cellulolyticum* is unable to metabolize high concentrations of cellobiose [25].

Also, degree of synergism (DS), defined as the activity of a mixture of components divided by the sum of the component activities evaluated separately [26], in this co-culture can be estimated as the ratio of *C. cellulolyticum* growth rates in co-culture and mono-culture under pH profile, and was equal to 7.99; although the co-culture DS determined based on the cellulolytic activities in the co-culture and the mono-culture would be substantially high, which indicates the presence of a strong synergism in this clostridial co-culture. The DS value of 5 or higher is not very common in enzyme-microbe cellulose hydrolysis systems and has been observed under some conditions [27,28]. Moreover, in this co-culture on fibrous cellulose, observed maximum cellulose degradation rate of 0.108 g/(L. h) is comparable with cellulose degradation rate of 0.15 g/(L. h) in *C. thermocellum* culture on crystalline cellulose, which shows one of the highest cellulose utilization rates among cellulolytic microorganisms [26].

Since cellulolytic bacteria are unable to grow at low intracellular pH, under acidic environment the pH gradient (ΔpH) across the cell membrane is high; consequently, the intracellular dissociation of fermentation acids, which are membrane permeable in undissociated form, and the intracellular accumulation of acid anions lead to anion toxicity, which is the likely reason of growth inhibition under acidic condition [29,30]. Furthermore, it has been shown that presence of lactate and acetate ions in an acidic medium leads to a significant decline of glutamate synthesis in *Clostridium sporogenes* MD1, which inhibits the bacterial growth [30]. Also, for *E. coli* culture at pH of 6, incubation of cells with 8 mM acetate for 20 min was shown to result in intracellular accumulation of acetate anions (240 mM), and reduced level of intracellular glutamate pools [31]. Furthermore, in mildly acidic *E. coli* cultures (pH of 6), inhibition of methionine biosynthesis by acetate (8 mM) and the toxicity of accumulated homocysteine have been indicated as the cause of growth inhibition by acetate under weak acid stress [32]. Addition of methionine to this culture can restore *E. coli* growth rate to some significant extent. This effect has been also reported for other organic acids.

In this clostridial co-culture, the synergy could be attributed to the exchange of some growth precursors and biomass constituents between *C. acetobutylicum* and *C. cellulolyticum*, which potentially enables the cellulolytic organism to grow and metabolize cellulose under acidic pH condition. *C. acetobutylicum* is a fermentative bacterium which is able to grow well under acidic conditions in acidogenic and solventogenic growth phases. The results of co-culture experiment under low concentration of *C. acetobutylicum*, which are presented in the next section, also provides support for the role of *C. acetobutylicum*.

### Experimental characterization of the co-culture metabolism with low concentration of *C. acetobutylicum*

**Figure 4:**
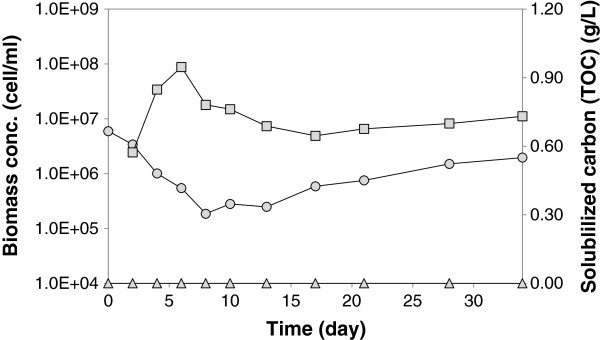
**The profiles of cellulose degradation and biomass formations in the co-culture experiments with low concentration of *C. acetobutylicum.*** The time profiles of *C. cellulolyticum* (circle), *C. acetobutylicum* (square) and cellulose solubilization in terms of total organic carbon (triangle) in the co-culture experiment with *C. acetobutylicum* inoculated after 2 days. The figure shows one data set representative of two independent experiments, with both showing comparable results.

To investigate if high initial concentration of *C. acetobutylicum* contributes to its low growth in the co-culture, and if *C. acetobutylicum* growth in the co-culture is affected by the ratio of cells to the cellulose hydrolysate, a co-culture experiment was conducted with 100 times lower initial concentration of *C. acetobutylicum*. 2 mL of *C. acetobutylicum* culture at exponential growth phase was centrifuged and the cell pellets were suspended in 2 mL of CGM medium and incoculated into the bioreactor. The results of this experiment are presented in Figure 4, where no cellulose degradation was observed in either biological replicates. Also, *C. cellulolyticum* as well as *C. acetobutylicum* did not grow in the co-culture*.* This experiment confirmed that the role of *C. acetobutylicum* in the metabolism of cellulose by the co-culture is associated with the population of *C. acetobutylicum*, and can be attributed to the exchange of some metabolites between the two species.

Furthermore, the metabolic behavior of *C. acetobutylicum* under the co-culture conditions was investigated (Additional file 1), where this study confirmed the metabolism of pyruvate and the released sugars by *C. acetobutylicum* in the clostridial co-culture, and that the observed oscillations in the *C. acetobutylicum* concentration in the co-cultures could be due to the slow release of sugars by *C. cellulolyticum* that can lead to starvation cycles for *C. acetobutylicum* in the co-culture.

### Analysis of product formations in the co-culture

**Figure 5:**
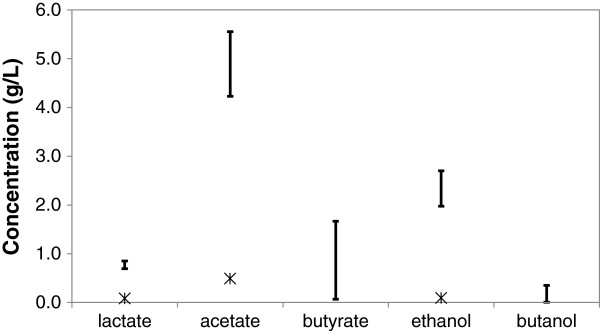
**Product concentrations in the co-cultures and the mono-culture under pH profile.** Product concentration ranges in the co-culture experiment A after 28 days (ranges have been calculated based on two sets of experimental data), and the product concentrations in the mono-culture batch under pH profile after 28 days (asterisk).

Figure 5 shows the ranges for co-culture and mono-culture product concentrations after 28 days. As it can be noted, acetate, ethanol, lactate, butyrate, and butanol were the main products of the fermentation in the co-culture. Butyrate appeared after *C. acetobutylicum* inoculation in the co-culture, but its concentration remained low. Neither acetate nor butyrate uptake, which are the characteristics of the solventogenic phase in *C. acetobutylicum* metabolism, was observed in this co-culture. At high cellulose concentration, *C. cellulolyticum* produces lactate as its main product along with acetate and ethanol [22]. The lactate uptake, observed in the co-culture batches, coincided with butanol formation (Additional file 1). The lactate uptake can be related to *C. acetobutylicum* metabolic activity, as *C. acetobutylicum* ATCC824 has been shown to co-ferment lactate and glucose [33].

It has been previously shown [22] that in pH controlled batch cultures of *C. cellulolyticum* on a defined medium, the distribution of carbon flow depends on the initial cellulose concentration. For concentrations less than 6.7 g/L of cellulose, acetate, ethanol, CO_2_ and H_2_ were shown to be the main fermentation end products and more than 91% of cellulose was observed to be degraded. At higher cellulose concentrations, from 6.7 g/L up to 29.1 g/L, carbon flow is redirected from ethanol and acetate towards lactate and extracellular pyruvate. In addition, in batch cultures of *C. cellulolyticum* on high cellulose concentration, it has been shown that the peak of pyruvate formation coincides with the start of lactate formation, and this pyruvate accumulation in the *C. cellulolyticum* culture shows that the rate of cellulose catabolism is higher than the rate of pyruvate consumption. Also it has been suggested that the cellulose hydrolysis depends on the concentration of *C. cellulolyticum*, which remains constant at and above 6.7 g/L of cellulose [22].

Furthermore, it has been shown that re-inoculating a fresh culture of *C. cellulolyticum* at high cellulose concentration of 29.1 g/L, where substrate is not fully consumed, significantly improves the cellulose solubilization and biomass yield compared to a classical batch [22]. This result indicates that the incomplete cellulose catabolism is not due to either the limitation of adhesion sites on cellulose fibers or product inhibition. At high cellulose concentrations, the likely explanation for the incomplete cellulose consumption is the lack of control on carbon uptake flow and an imbalanced metabolism leading to the accumulation of intracellular metabolites and self-intoxication of the cells, eventually resulting in a growth arrest [22,34]. Similarly, extracellular pyruvate formation has been reported in *C. thermocellum* cultures at high cellulose and cellobiose concentrations, which evidences the overflow metabolism [35].

In our experiments, the maximum concentration of *C. cellulolyticum* in co-culture experiments was the same as the mono-culture experiment under optimal pH of 7.2, however the cellulose degradation was improved up to 82% (Figure 3c), which confirms the role of *C. acetobutylicum* in cellulose degradation, while *C. cellulolyticum* has reached the stationary growth phase. We observed pyruvate accumulation of 0.029 g/L in the mono-culture batch under the co-culture pH profile and 0.004 g/L in the mono-culture batch at pH of 7.2. In the co-culture replicates, maximum pyruvate concentration of 0.17 g/L was observed, which was taken up later during the course of experiments coinciding with butyrate formation in the co-cultures. Our previous modeling studies have suggested that limited pyruvate-ferredoxin oxidoreductase (PFO) activity, which cannot support high pyruvate flow, results in pyruvate overflow [36]. Hence, a potential explanation for pyruvate secretion in *C. cellulolyticum* cultures is the limitation on the pyruvate consumption rate and a comparatively higher carbon catabolism rate, and due to inefficient regulation of entering carbon flow [25]. Furthermore, intracellular pyruvate accumulation could be the explanation for the growth arrest at high cellulose concentrations [37], at which cells switch to stationary growth phase before substrate depletion.

Pyruvate uptake in the co-culture can be explained by the capability of *C. acetobutylicum* to metabolize pyruvate. It has been also reported that providing *C. acetobutylicum* with pyruvate as the sole carbon source results in the growth and production of mainly acetate and butyrate [38]. In another co-culture study, the removal of *C. cellulolyticum* metabolic products such as pyruvate and their associated inhibitory effects, by *Rhodopseudomonas palustris* in the co-culture of *C. cellulolyticum* and *R. palustris* has been reported as the underlying reason for the improved cellulose degradation and bacterial growth in this co-culture [39]. *C. cellulolyticum* growth on cellulose has been shown to be severely inhibited by pyruvate; where about 60% decrease in the biomass concentration in the presence of 2 mM (176 mg/L) pyruvate has been observed in *C. cellulolyticum* mono-culture [39]. Therefore, pyruvate removal by *C. acetobutylicum* and alleviating its inhibitory effect can be a contributing factor in the improved growth of *C. cellulolyticum* and its boosted cellulolytic activity in the co-culture.

The major products of pyruvate fermentation by *C. acetobutylicum* are acetate, butyrate and butanol, and neither acetate nor butyrate is reutilized. The effects of pyruvate on glucose fermentation by *C. acetobutylicum* have also been investigated before, and it has been shown that both substrates can be fermented simultaneously [40]. Furthermore, cellobiose and glucose were only detected at the early stage of batches, which could have been present in the pre-cultures, inoculated into the bioreactors, and were taken up in 24 hours. Cellobiose and glucose could not be detected in the course of co-cultures which indicated their immediate consumption in the co-culture. In conclusion, in this study we showed a strong synergism between the two species of clostridia in the co-culture, and found that *C. acetobutylicum* enables *C. cellulolyticum* to grow under harsh co-culture environment. This synergy can be attributed to the production of some growth pre-cursors, and future metabolomic studies of this co-culture can identify such metabolites.

## Conclusions

Examining of the metabolism in this clostridial co-culture along with the mono-cultures revealed that significant increase in the rate of cellulose hydrolysis can be achieved using the co-culture and making use of the synergism existing between the two clostridial species. It is likely that *C. acetobutylicum* improves the cellulolytic activity of *C. cellulolyticum* in the co-culture through exchange of metabolites such as pyruvate, enabling it to grow and metabolize cellulose under harsh co-culture conditions. This clostridial co-culture can offer a considerable potential CBP approach for producing commodity chemicals from cellulosic biomass, taking advantage of *C. acetobutylicum* metabolic potential in converting sugars to variety of chemicals.

## Methods

### Strains and media

*C. acetobutylicum* ATCC 824 was grown in clostridial growth medium (CGM) containing per litre: 0.75 g of KH_2_PO_4_, 0.75 g of K_2_HPO_4_, 1 g of NaCl, 0.01 g of MnSO_4_, 0.004 g of *p*-aminobenzoic acid, 0.348 g of MgSO_4_, 0.01 g of FeSO_4_, 2 g of asparagine, 5 g of yeast extract, 2 g of (NH_4_)_2_SO_4_ and 50 g of glucose (pH 5.8). *C. cellulolyticum* ATCC 35317 was maintained and cultivated on a modified CM3 medium containing per litre: KH_2_PO_4_, 1.5 g; K_2_HPO_4_, 2.9 g; (NH_4_)_2_SO4, 1.3 g; MgC1_2_.6H_2_O, 1 g; CaC1_2_, 0.15g; FeSO_4_, 1.25 mg; resazurin, 1 mg; cysteine hydrochloride, 1 g; fibrous cellulose (Sigma-Aldrich, C6288), 7.5 g, and 2 g of yeast extract (pH 7.2).

The co-culture medium was composed of 0.75 g of KH_2_PO_4_, 1 g of MgC1_2_.6H_2_O, 0.15 g of CaC1_2_, 1 mg of resazurin, 1 g of cysteine hydrochloride, 0.01 g of MnSO_4_, 0.004 g of *p*-aminobenzoic acid, 10 mg of (NH_4_)_2_Mo_7_O_24_, 0.01 g of FeSO_4_, 2 g of asparagine, 3 g of yeast extract, 1 g of (NH_4_)_2_SO_4_ and 20 g of fibrous cellulose per litre of medium. All pre-cultures were incubated at 37°C without shaking in serum bottles to reach the total protein concentration of 15 to 20 mg/L (Bio-Rad Protein Assay, 500–0006).

### Co-culture experiments

All batch cultivations were conducted in 5 L bioreactors with a working volume of 2 L at 37°C, where agitation was set at 200 rpm, and the volume of *C. cellulolyticum* inoculum at exponential growth phase formed about 10% of the total volume. The *C. cellulolyticum* pre-cultures were transferred twice on the co-culture medium with 7.5 g/l of cellulose before inoculation into bioreactors, where the biomass concentration in the bioreactor inocula was between 6×10^8^ and 8×10^8^ cell/mL. For the co-culture batches, *C. cellulolyticum* was first cultivated for 48 hours at pH 7.0; then 200 mL of *C. acetobutylicum* culture at exponential phase was centrifuged and the cell pellets were suspended in 20 mL of CGM medium and inoculated into the bioreactor. The pH then was adjusted and maintained at 6.0 using 1N H_2_SO_4_ and 3N NaOH. The bioreactors were kept under anaerobic conditions by continuous sparging of nitrogen gas. The same pH adjustment and medium were applied for the *C. cellulolyticum* mono-culture batch unless for the mono-culture batch at pH 7.2 that was conducted on CM3 medium with no pH profile.

### Analysis

Concentration of sugars, organic acids and alcohols were measured using Dionex Ultimate 3000 HPLC (Bio-Rad, Hercules, CA) equipped with a UV monitor (210 nm) and a Shodex RI-101 differential refractive index detector. The products were separated using a Bio-Rad HPX-87H cation-exchange column, where 10 mM H_2_SO_4_ was used as the mobile phase (0.6 mL/min flow rate, 60°C column temperature, 20 μL injection volume). To account for the extracellular proteins, amino acids and amino compounds in cell cultures [36], and to estimate the overall carbon recovery, total organic carbon (TOC) concentrations of the sample supernatants were quantified using Total Organic Carbon Analyzer (TOC-V_CPH/CPV_, Shimadzu, Japan), with the lower detection limit of about 0.5 mg/L using standard TOC catalyst. Cellulose concentration was measured using Updegraff method [41]. Cellulose pellets were washed using acetic acid-nitric acid reagent and water to remove the non-cellulosic material; cellulose then was quantified using anthron in a colorimetric method. The results of cellulose assay and TOC measurements showed that about 24% of the total carbon was used for CO_2_ formation, which was consistent with our previous model prediction [42].

To measure protein, 0.5 ml of cell culture was centrifuged at 8,000 *g* for 2 min and washed with 0.9% (wt/vol) NaCl. The pellet was resuspended in 0.5 ml of 0.2 N NaOH, and this suspension was placed in a boiling water bath for 10 min. After cooling, the hydrolyzed sample was centrifuged as described above, and the total solubilized protein concentration in supernatants was measured using Bio-Rad Protein Assay (500–0006). Quantification of the population of each species in the co-culture was conducted using quantitative PCR (qPCR) method, which includes DNA extraction, PCR amplification of 16SrRNA gene and detection using fluorescent dyes, and is elaborated in following sections.

#### Primer design and qPCR standard plasmid DNA preparation

The qPCR primers were designed to target the 16SrRNA genes in *C. acetobutylicum* (CA_Cr001) and *C. cellulolyticum* (Ccel_R0007) as reported in Table 1. The qPCR standard plasmid solutions for each target species were prepared by cloning the purified PCR products into pCR®2.1-TOPO® vector using TOPO-TA Cloning® kit (Invitrogen™, K4500-01). In the first step, fresh PCR products were purified using GeneJET PCR Purification Kit (Fermentas, K0701,K0702); the nucleic acid concentrations in purified PCR products were measured to be 67 and 50 ng/μl for *C. acetobutylicum* and *C. cellulolyticum*, respectively, using NanoDrop 1000 (Thermo Scientific) spectrophotometer, and PCR products purity were verified using agarose gel electrophoresis. Plasmid extraction from the positive clones was conducted using GenElute Plasmid Miniprep kit (Sigma-Aldrich, USA). All of the extracted plasmids were sequenced at the University of Toronto Sanger Sequencing Facility, and the plasmids with right inserts were selected and applied for qPCR calibrations.

**Table 1:** Primer sequences used for qPCR analysis

**Primer name**	**Target species**	**Primer sequence (5′-3′)**	**Product length**	**T**_**m**_
CA2 (forward)	*C. acetobutylicum*	CTTGTTGGTGAGGTAACGG	386 bp	60°C
CA2 (reverse)	*C. acetobutylicum*	CACTCCAGACATCCAGTTTG		
CC2 (forward)	*C. cellulolyticum*	TACAGGGGGATAACACAGG	348 bp	60°C
CC2 (reverse)	*C. cellulolyticum*	CGTGGCTTATTCTTCAGGTAC		

There are eleven 16srRNA genes in *C. acetobutylicum* genome (CA_Cr001, CA_Cr004, CA_Cr007, CA_Cr010, CA_Cr013, CA_Cr016, CA_Cr019, CA_Cr022, CA_Cr025, CA_Cr028, CA_Cr033), and eight 16srRNA genes in *C. cellulolyticum genome* (Ccel_R0007, Ccel_R0018, Ccel_R0088, Ccel_R0084, Ccel_R0059, Ccel_R0012, Ccel_R0024, Ccel_R0036). Multiple sequence alignment of the 16srRNA genes for each species using ClustalW2 [43] showed identical amplification regions for each set of 16SrRNA genes, therefore 8 amplicons with the same size are being produced per copy of *C. cellulolyticum* genome in a qPCR reaction, and 11 amplicons with the same size are being produced per copy of *C. acetobutylicum* genome in a qPCR reaction; these facts also were verified by observing single peaks in melting curve analysis as well as single DNA bands for each species in agarose gel electrophoresis runs, and must be considered in qPCR quantifications of each species. For each qPCR run a new standard curve was made using fresh dilutions, where the standard curve concentrations of 10^8^ copies/μL to 10^1^ copies/μL were prepared by making serial 1:10 dilutions starting with the 10^9^ copies/μL plasmid solution.

#### Genomic DNA isolation

Initially the Mo Bio Laboratories UltraClean® Soil DNA Isolation Kit was applied to extract DNA from the samples. However, it was demonstrated that there was no correlation between culture size and the DNA yield using this kit for co-culture samples, as the same amount of DNA was extracted from 0.2, 1, 2 and 5 mL of cultures; in fact this kit has been aimed for soil samples where the concentrations of biomass is typically low, and therefore there was a chance to miss significant amount of DNA in the co-culture samples. Consequently, DNA isolation using Norgen bacterial genomic DNA isolation kit (Norgen, #17900) was tested, and it was found that there was a correlation between the sample size (in the range of 0.2 to 0.5 mL) and DNA yield using Norgen kit, while it had a higher DNA yield compared to the Mo Bio kit. Therefore, the Norgen kit was chosen for DNA isolations; culture samples (0.5 mL) were centrifuged at 13000 g for 2 minutes, and the cell pellets were used for DNA isolations following the standard kit protocol, where 400 μL elution buffer was used lastly to elute DNA in two steps (200 μL for each dilution step).

#### qPCR reaction preparation, detection and quantification

The qPCR amplification was performed using 2 μL of tenfold diluted sample genomic DNA and 18 μL of a master mix in a total reaction volume of 20 μL. A master mix was prepared for each qPCR run whereby the reagents for the individual reactions were combined prior to plate preparation in order to minimize errors. Master mixes were prepared by combining 10 μL of SsoFast™ EvaGreen® Supermix (Bio-Rad, #172-5200), 1 μL of each primer with final concentration of 0.25 μM, and 6 μL of water for each reaction, and 2 μL of DNA solution was added to each reaction in a 20 μL reaction volume.

The qPCR amplifications and detections were carried out in a CFX96™ Real-Time PCR Detection System (Bio-Rad Laboratories Inc.). The qPCR program had the following protocol: 1) initial denaturation at 98°C for 3 min, 2) 40 cycles of denaturation at 98°C for 5 sec, 3) annealing and extension at 60°C for 10 sec followed by a plate read; afterward a melting curve analysis from 65 to 95°C measuring fluorescence every 0.5°C. For all qPCR runs, the qPCR signals were analyzed at a standard pre-determined threshold of 1E+03 RFU which was in the exponential amplification phase and above the background fluorescence noise for all the qPCR runs. The quantification cycles (C_q_ or C_T_), the cycle number where fluorescence increases above the threshold, were used to find the DNA copy numbers (automatically calculated from the standard curve). To examine the quality of qPCR assays, standard curve with coefficient of determination (R^2^) > 0.980, high amplification efficiency (90–110%), and consistency across replicate reactions were considered.

## Abbreviations

TOC: Total organic carbon; CBP: Consolidated bioprocessing; DS: Degree of synergism.

## Competing interests

The authors declare that they do not have any competing interests.

## Authors’ contributions

FS conceived of the study, designed and conducted the experiments, and drafted the manuscript. RM conceived of the study and helped to draft the manuscript. Both authors read and approved the final manuscript.

## Supplementary Material

Additional file 1: Figure S1 (a) Time profiles of lactate (triangle), acetate (strike), butyrate (circle) and ethanol (rhombus), and (b) time profiles of pyruvate (triangle) and butanol (rhombus) in the co-culture experiment A. Pyruvate uptake and butanol formation were observed in the co-cultures. **Figure S2.***C. acetobutylicum* biomass profiles in the mono-cultures under co-culture conditions. (a) *C. acetobutylicum* was cultivated on co-culture medium at pH of 6.0 and 20 g/L cellulose without glucose/pyruvate addition, and characterized using qPCR (rhombus) and hemacytometry (square). (b) *C. acetobutylicum* culture on co-culture medium at pH of 6.0 and 20 g/L cellulose. 1 g/L glucose was added at day 12, and 1 g/L pyruvate was added to the culture on day 19. Click here for file
