# Supplementary material for: Characterizing metabolic interactions in a clostridial co-culture for consolidated bioprocessing
Source: BMC Biotechnol. 2013 Nov 4;13:95. doi: 10.1186/1472-6750-13-95 (PMC3827933; doi:10.1186/1472-6750-13-95)
Supplement: Additional file 1: Figure S1 — (a) Time profiles of lactate (triangle), acetate (strike), butyrate (circle) and ethanol (rhombus), and (b) time profiles of pyruvate (triangle) and butanol (rhombus) in the co-culture experiment A. Pyruvate uptake and butanol formation were observed in the co-cultures. Figure S2.C. acetobutylicum biomass profiles in the mono-cultures under co-culture conditions. (a) C. acetobutylicum was cultivated on co-culture medium at pH of 6.0 and 20 g/L cellulose without glucose/pyruvate addition, and characterized using qPCR (rhombus) and hemacytometry (square). (b) C. acetobutylicum culture on co-culture medium at pH of 6.0 and 20 g/L cellulose. 1 g/L glucose was added at day 12, and 1 g/L pyruvate was added to the culture on day 19. [file 1472-6750-13-95-S1.docx]

**Supplementary Figure 1.** (a) Time profiles of lactate (triangle), acetate (strike), butyrate (circle) and ethanol (rhombus), and (b) time profiles of pyruvate (triangle) and butanol (rhombus) in the co-culture experiment set A. Pyruvate uptake and butanol formation were observed in the co-cultures.

## Experimental Characterization of *C. acetobutylicum* Metabolic Activity under Co-culture Conditions

To further investigate the metabolic behavior and the role of *C. acetobutylicum* in the clostridial co-culture, mono-culture control batches of *C. acetobutylicum* were conducted under the co-culture conditions. The results of these experiments are presented in below Figs. 2a and b. In the first mono-culture batch (Fig. 2a), *C. acetobutylicum* was cultivated on the co-culture medium with 20 g/L cellulose and no glucose added to the culture. The qPCR data as well as cell quantification using hemacytometry showed a significant reduction in the cell population over the course of batch due to the lack of glucose and cell starvation.

In the second mono-culture batch (Fig. 2b), after 12 days of cultivation and cell starvation in the absence of glucose, 1 g/L of glucose was added to the culture and as a result a drastic increase in the biomass concentration was observed in the culture followed by a decline due to cell starvation after consuming the entire added glucose in 24 hrs. Furthermore, to assess *C. acetobutylicum* growth and metabolism on pyruvate, 1 g/L of pyruvate was added to the culture on day 19^th^, while the cells had been starving for previous 6 days, and a 10-fold increase in the biomass concentration was observed, confirming metabolism of pyruvate by *C. acetobutylicum*. However, pyruvate was not consumed entirely (HPLC data showed that only about 0.1 g/L of pyruvate had been consumed), and the main fermentation products were acetate and butyrate. Higher biomass concentration obtained from qPCR method can be attributed to the spore formation in *C. acetobutylicum* cultures, as both viable and non-viable spores in addition to dead cells are quantified using q-PCR [1] in contrast to hemacytometry. Also, the similar trends observed in the biomass profiles using both qPCR and hemacytometry methods verified the appropriateness of the developed qPCR method. This study confirmed the metabolism of pyruvate and the released sugars by *C. acetobutylicum* in the clostridial co-culture, and that the observed oscillations in the *C. acetobutylicum* concentration in the co-cultures could be due to the slow release of sugars by *C. cellulolyticum* that can lead to starvation cycles for *C. acetobutylicum* in the co-culture.

Supplementary Figure 2. *C. acetobutylicum* biomass profiles in the mono-cultures under co-culture conditions. (a) *C. acetobutylicum* was cultivated on co-culture medium at pH of 6.0 and 20 g/L cellulose without glucose/pyruvate addition, and characterized using qPCR (rhombus) and hemacytometry (square). (b) *C. acetobutylicum* culture on co-culture medium at pH of 6.0 and 20 g/L cellulose. 1 g/L glucose was added at day 12, and 1 g/L pyruvate was added to the culture on day 19.

Reference:

1. Rawsthorne H Fau - Dock CN, Dock Cn Fau - Jaykus LA, Jaykus LA: **PCR-based method using propidium monoazide to distinguish viable from nonviable Bacillus subtilis spores.** *Applied and Environmental Microbiology* 2009, **75:**2936–2939.
